# Neonatal Intermittent Hypoxia Induces Lasting Sex-Specific Augmentation of Rat Microglial Cytokine Expression

**DOI:** 10.3389/fimmu.2019.01479

**Published:** 2019-07-02

**Authors:** Elizabeth A. Kiernan, Tao Wang, Amanda M. Vanderplow, Sneha Cherukuri, Michael E. Cahill, Jyoti J. Watters

**Affiliations:** ^1^Neuroscience Training Program, University of Wisconsin-Madison, Madison, WI, United States; ^2^Department of Comparative Biosciences, University of Wisconsin-Madison, Madison, WI, United States

**Keywords:** microglia, intermittent hypoxia, development, neonate, sex differences

## Abstract

Sleep disordered breathing (SDB) affects 3–5% of the pediatric population, including neonates who are highly susceptible due to an underdeveloped ventilatory control system, and REM-dominated sleep. Although pediatric SDB is associated with poor cognitive outcomes, very little research has focused on models of pediatric SDB, particularly in neonates. In adults and neonates, intermittent hypoxia (IH), a hallmark of SDB, recapitulates multiple physiological aspects of severe SDB, including neuronal apoptosis, sex-specific cognitive deficits, and neuroinflammation. Microglia, resident CNS immune cells, are important mediators of neurodevelopment and neuroinflammation, but to date, no studies have examined the molecular properties of microglia in the context of neonatal IH. Here, we tested the hypothesis that neonatal IH will enhance microglial inflammation and sex-specifically lead to long-term changes in working memory. To test this hypothesis, we exposed post-natal day (P1) neonates with dams to an established adult model of pathological IH consisting of 2 min cycles of 10.5% O_2_ followed by 21% O_2_, 8 h/day for 8 days. We then challenged the offspring with bacterial lipopolysaccharide (LPS) at P9 or at 6–8 weeks of age and immunomagnetically isolated microglia for gene expression analyses and RNA-sequencing. We also characterized neonatal CNS myeloid cell populations by flow cytometry analyses. Lastly, we examined working memory performance using a Y-maze in the young adults. Contrary to our hypothesis, we found that neonatal IH acutely augmented basal levels of microglial anti-inflammatory cytokines, attenuated microglial responses to LPS, and sex-specifically altered CNS myeloid populations. We identified multiple sex differences in basal neonatal microglial expression of genes related to chemotaxis, cognition, and aging. Lastly, we found that basal, but not LPS-induced, anti-inflammatory cytokines were augmented sex-specifically in the young adults, and that there was a significant interaction between sex and IH on basal working memory. Our results support the idea that neonates may be able to adapt to IH exposures that are pathological in adults. Further, they suggest that male and female microglial responses to IH are sex-specific, and that these sex differences in basal microglial gene expression may contribute to sexual dimorphisms in vulnerability to IH-induced cognitive disruption.

## Introduction

Obstructive sleep apnea (OSA), a form of sleep disordered breathing (SDB) characterized by repetitive episodes of intermittent hypoxia (IH), is present in up to 5% of the total pediatric population including infants (1). Further, up to 80% of infants with craniofacial abnormalities, such as those associated with Down Syndrome will present with OSA (2), and some evidence suggests a higher occurrence in males (3). Despite the common occurrence of OSA in infants, it remains poorly characterized in terms of clinical presentation, partly due to its co-morbidity with other early life complications (4, 5). In addition to the poorly characterized clinical presentations, the long-term effects of infant OSA are unknown. In older pediatric populations, OSA is associated with poor behavioral outcomes and neurocognitive dysfunction (6). Although vulnerable infant populations (i.e., preterm or low-birthweight) are at risk for OSA (7), systemic infection (8), and adverse neurodevelopment (9), little is known about how OSA contributes to these co-morbid processes.

Rodent models of OSA suggest IH alone recapitulates multiple deficits that characterize OSA, including neuronal apoptosis (10), peripheral and central inflammation (11, 12), and cognitive deficits (13). In neonates, long term exposure to IH causes lower weight gain (14), hippocampal apoptosis (15), hypomyelination (16), changes in sympathetic nerve activity (17–19) and attenuation of adult male spatial memory (10). Additionally, neonatal IH enhances the male, but not female, hypoxic ventilatory response (20). Thus, IH recapitulates some of the sex-differences observed in human neonatal populations, and suggests that similar to the findings for preterm infants [reviewed in (21)], males may be a particularly vulnerable population.

Microglia, CNS resident macrophages, are important mediators of both neuroinflammation and neurodevelopmental processes (22–28), including learning and memory (29). Further, changes in microglia morphology and/or function are associated with abnormal neurodevelopment (30–32). Preterm infants, who are susceptible to both OSA (7) and long-term cognitive dysfunction (33), have altered microglia accumulation at sites of white matter injury (34). In adult rats, IH augments microglial expression of classic inflammatory molecules such as toll-like receptor 4 (TLR4), cyclooxygenase-2 (COX-2), inducible nitrogen oxide synthase (iNOS), and interleukin-1 (IL-1) (11), and attenuation of inflammation using inhibitors of COX-2 and iNOS improves cognitive outcomes (13, 35). While pediatric OSA and neonatal IH are associated with systemic and carotid body inflammation (19, 36–38), it is unknown if OSA or IH alters microglial inflammation during postnatal development, or if this is associated with differences in cognitive outcomes.

Similar to neonatal hypoxic responses, there are developmental sex differences in microglia, including their morphology (39) and dynamics (40), which, when perturbed, may account for the sex differences in neurodevelopmental disorders (41) [reviewed in (42)]. The phagocytic capacity of microglia is also sexually dimorphic, with female microglia phagocytosing more neural progenitor cells in the early postnatal period (43). Although not yet directly linked to a microglial source, brain expression of key inflammatory cytokines, such as interleukin-1β, and C-x-c motif cluster inflammatory chemokines including Cxcl9, Cxcl10, and Cxcl11, also differs between males and females in the neonatal rat (39), suggesting basal sex differences in microglial inflammatory responses. The sex-specific effects of neonatal IH on microglial expression of these immune related genes has never been tested.

We thus hypothesized that neonatal IH would sex-specifically augment microglial pro-inflammatory gene expression both acutely and long-term, and correlate with long-term deficits in working memory. Surprisingly, we found that neonatal IH enhanced gene expression related to type I interferon responses and “anti-inflammatory” signaling in both males and females, effects which persisted long-term in males. Additionally, in the presence of systemic inflammation, IH-exposed females, but not males had attenuated cytokine expression, and enrichment of a putative CNS monocyte-derived population. RNAseq analyses of male and female microglia confirmed these results and identified sex differences in genes related to leukocyte chemotaxis and cognition. Finally, behavioral analyses of adults exposed to IH as neonates demonstrated that while there was a significant interaction between the effects of IH and sex, neonatal IH had no effect on working memory.

## Methods

### Animals and Intermittent Hypoxia (IH) Exposures

All animal experimental procedures were performed according to the NIH guidelines set forth in the Guide for the Care and Use of Laboratory Animals and were approved by the University of Wisconsin-Madison Institutional Animal Care and Use Committee. Timed-pregnant Sprague-Dawley rats (E16) were obtained from Charles River (RRID:RGD_734476; Wilmington, MA) and housed in AAALAC-accredited facilities with 12 h:12 h light–dark conditions. Food and water were provided *ad libitum*. Beginning at postnatal day (P) 1, dams with neonates were exposed to 8 days of an IH paradigm which consisted of alternating 2-min hypoxic (45 s down to 10.5% O_2_) and normoxic (15 s up to 21% O_2_) episodes for 8 h/day (9:00 am−5:00 pm) as previously reported (44). This produces an apnea hypopnea index (AHI) of 15 events/h which mimics kinetics of moderate sleep apnea in pediatric populations and adults (45). Normoxic (Nx) controls received alternating room air. At ~16 h or 6 weeks following the final IH episode, animals were weighed and injected intraperitoneally with either HBSS vehicle (Cellgro; Herndon, VA) or a sublethal 0.1 mg/kg lipopolysaccharide (LPS E. coli O11:B4; 500,000 EU/mg; Sigma-Aldrich; St. Louis, MO) for 3 h. Following LPS, pups were sacrificed via decapitation and brains isolated in HBSS for removal of meninges and blood. Pulse-oximetry was performed at P1 on sentinel rats (*n* = 4) using STARR MouseOx Rev 6.3.13; these rats were excluded from microglial analyses.

### Microglia Isolation

CD11b/c^+^ cells were immunomagnetically isolated from the whole brain (minus the cerebellum and olfactory bulbs) as previously described (11) using the Miltenyi OX-42 antibody (Miltenyi Biotec Inc., Auburn, CA Cat# 130-105-634). Myelin was removed using 26% Percoll (GE HealthCare; Madison, WI). Cells were then either resuspended in a modified zinc-based fixative (mZBF) for subsequent flow cytometry analyses (46) or column separated according to the Miltenyi MACs protocol. Column separation results in 97.9% viable cells and 88–96% of CD11b^+^ cells were microglia (CD11b/CD45^low^) (47). Isolated CD11b^+^ cells were resuspended in TriReagent (Invitrogen, Carlsbad, CA), and total RNA extracted according to the manufacturer's instructions.

### Quantitative RT-PCR

cDNA was synthesized as previously described (48). MMLV Reverse Transcriptase was purchased from Invitrogen. Oligo dT, Random Primers, and RNAse inhibitor were purchased from Promega (Madison, WI). qRT-PCR using power SYBR green (ThermoFisher Scientific) was performed as previously described using an Applied Biosystems 7500 Fast Real Time PCR System (11). All primers (Table 1) were tested for efficiency using serial dilutions, and results were normalized to 18S RNA levels; data analyses were performed using the delta-delta CT method (49).

**Table 1:** Primers used for quantitative PCR.

**Gene target**	**Forward primer**	**Reverse primer**
*Inos*	GCTTGGGTCTTGTTAGCCTAGT	GTTGTTGGGCTGGGAATAGC
*Il1β*	GACTTCACCATGGAACCCGT	GGAGACTGCCCATTCTCGAC
*Ifnβ1*	GCTGAGGTTGAGCCTTCCAT	TGCCCTCTCCATCGACTACA
*Il1α*	CGCTTGAGTCGGCAAAGAAAT	CCTTGAAGGTGAAGGTGGACA
*Cxcl10*	CCGCATGTTGAGATCATTGCC	CTAGCCGCACACTGGGTAAA
*Il4*	GTTAGGACATGGAAGTGCAGGA	CGTGAATGAGTCCACGCTCA
*Cox2*	TGTTCCAACCCATGTCAAAA	CGTAGAATCCAGTCCGGGTA
*Stat3*	TGGAAAAGGACATCAGTGGCAAGA	TACCTGGGTCAGCTTCAGGGT
*18S*	CGGGTGCTCTTAGCTGAGTGTCCC	CTCGGGCCTGCTTTGAACAC

### PCR Gene Array

cDNA for microglia derived from male Nx LPS- and IH LPS-treated neonates was synthesized as for RT-PCR, aliquotted to the Qiagen (Hilden, Germany) NF-κB Signaling Targets RT^2^ Profiler PCR Array (cat # PARN-225Z) and run on the Applied Biosystems 7500 Fast Real Time PCR System. Results were analyzed using Qiagen software and exported to Microsoft Excel to generate heatmaps.

### Total RNA Sequencing and Bioinformatics Analyses

Total microglial RNA was isolated from neonatal pups exposed to 8 days of IH followed by vehicle or LPS challenge. Three biological replicates from each treatment were submitted to the UW-Madison Biotechnology Resource Center for sequencing and quality control. Stranded cDNA libraries were prepared using the Truseq Stranded mRNA kit (Illumina; San Diego, CA). Sequencing was performed using an Illumina HiSeq 2500. After sequencing and quality control, Fastq files containing ~10 million reads for each biological replicate (*n* = 3/treatment) were aligned using TopHat 2.1.0 with the following parameters: –no-novel-juncs, – Library type fr – firststrand. BAM files were converted to SAM with SAMtools. Read counts were obtained using HTSeq v. 0.6.1 with the stranded reverse option. Count files were imported to R and filtered such that only genes with a CPM >1 expressed in three samples were retained. Counts were normalized using the Trimmed Mean of M-Values method (TMM), and analyzed for differential expression using EdgeR (50, 51). Genes with an FDR < 0.05 were considered differentially expressed. Results were uploaded to NCBI Geo Datasets (GEO GSE126946). To specifically probe RNA-seq data for changes in NFκB signaling target genes obtained from the Qiagen PCR array (section PCR Gene Array), NFκB target genes were ranked by *p*-value and FDRs calculated for the specific set of genes. NFκB target genes with an FDR < 0.05 were considered differentially expressed. Gene ontology was performed on differentially expressed genes using both DAVID (52, 53) and Panther (54) for GO class Biological Process. For GO analyses, background files were generated using the filtered count files. Motif analysis of rat promoters for gene lists was performed using the find.Motifs.pl script in Homer (55). Protein-protein interaction networks were created using STRING (56).

### Flow Cytometry

Single cell suspensions of P9 whole brain homogenates were created by pushing the tissue through 40 μm filter with ice-cold HBSS supplemented with 0.01 mg/ml DNase and 0.1 mM EDTA. Myelin was removed by high-speed centrifugation at 850 g for 15 min in a solution consisting of 26% Percoll in 0.1 M PBS. Dissociated cells were washed in ice-cold HBSS and pelleted by centrifugation at 350 g for 5 min. Cells were resuspended in mZBF (0.5% zinc chloride, 0.5% zinc trifluoroacetate, 0.05% calcium acetate in 0.1 M Tris–HCL, pH 6.4–6.7) and glycerol (1:1) (46). Prior to fixation, cells were incubated with eFluor 780 live/dead (eBioscience cat # 65-0865-14). The following mouse anti-rat antibodies were used: CD11b/c-PE (BD Pharmingen cat # 554862; RRID:AB_395562), CD45-PE-Cy7 (BD Pharmingen cat # 561588; RRID:AB_10893200), and CD4-APC (BD Pharmingen cat # 550057; RRID:AB_398458). Flow cytometry was performed using a BD Fortessa Flow Cytometer (gating strategy, Supplementary Figure 1). FCS files were analyzed using FlowJO v.10.0.7 software.

### Spontaneous Alternation Test

The Y-maze is a measure of working memory and was performed as previously described (57, 58). Animals were between 6 and 8 weeks of age and behaviorally naïve prior to the start of testing. Arm entry was quantified when an animal had its hind paws completely within a maze arm. Animals were placed into the Y-maze and allowed 15 min to explore. Analysis of arm entries began after the animal entered each arm once and arm choices were recorded for 10 min. The number of times 3 successive arm alternations were made correctly (clockwise or counterclockwise) was summed and then divided by the total number of arm entries minus two. The resulting number is the % of spontaneous alternations, with 50% being considered chance levels.

### Statistical Analyses

All statistical analyses were performed using SigmaPlot (Systat Software, San Jose CA) unless otherwise noted. Data that were not normally distributed were natural log transformed. The Grubb's Test was used to identify outliers. Statistical significance (set at *p* < 0.05) was determined using a Two-way analysis of variance (Two-way ANOVA) or Student's *t*-test, followed by a Holm-Sidak *post hoc* test when applicable. *P*-values between 0.05 and 0.1 were considered to be statistical trends.

## Results

### Neonatal IH for 8 Days Reduces SpO_2_ and Weight in Both Males and Females and Increases CNS Cell Death

**Figure 1:**
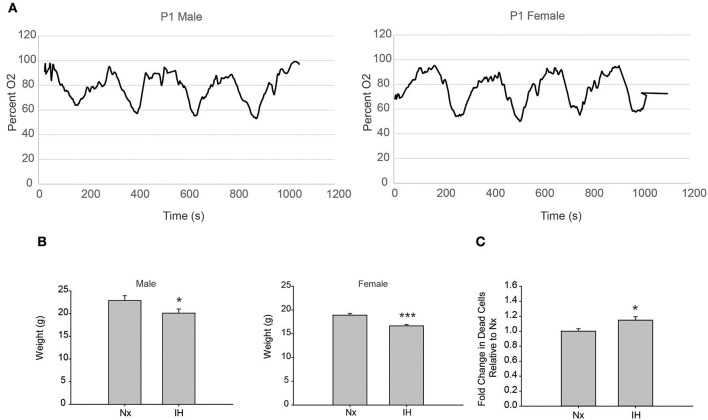
Neonatal IH decreases SpO_2_ and weight. **(A)** Pulse-oximetry was recorded in P1 neonatal males (left) and females (right). **(B)** At P9, after 8 days of Nx or IH exposure, male (left; *n* = 20/tx) and female (right; *n* = 20/tx) pups were weighed. **(C)** At P9 after 8 days of Nx or IH exposures, whole brains from male (*n* = 6/treatment) and female (*n* = 6/treatment) pups were isolated for flow cytometric analysis of cell death. *p < 0.05, ***p < 0.001, *t*-test.

To test the effects of 8 days of neonatal IH on microglial gene expression, we used a model of IH that is used in adult rat microglial studies (11) and is similar to models used in neonate studies (14, 38). We recorded blood oxygen saturation (SpO_2_) using pulse-oximetry at P1 (Figure 1A). Weights were recorded after 8 days of Nx or IH, just prior to LPS injections (Figure 1B). IH reduced SpO_2_ to just below 60% in both males and females. Additionally, pups exposed to 8 days of IH weighed less than their normoxic counterparts by P9; this was true for both males (*p* = 0.032, *n* = 20/tx) and females (*p* < 0.001, *n* = 20/tx) and is consistent with previous observations (14). In a separate cohort of animals, whole brains were isolated to assess cell death by flow cytometric analysis (Figure 1C). Using the live/dead dye eFluor 780, we found that IH significantly increased the percent of labeled cells (*p* = 0.017, *n* = 12/tx, mixed sex), indicating that IH induces neonatal CNS cell death. Thus, this model of IH which mimics moderate OSA and an infant apnea-hypopnea index (AHI) of 15 (45) causes blood O_2_ desaturations, reduces weight gain, and increases CNS cell death.

### Neonatal IH Sex-Specifically Upregulates Microglial Anti-inflammatory Cytokine and Attenuates LPS-Induced Cytokine Gene Expression

**Figure 2:**
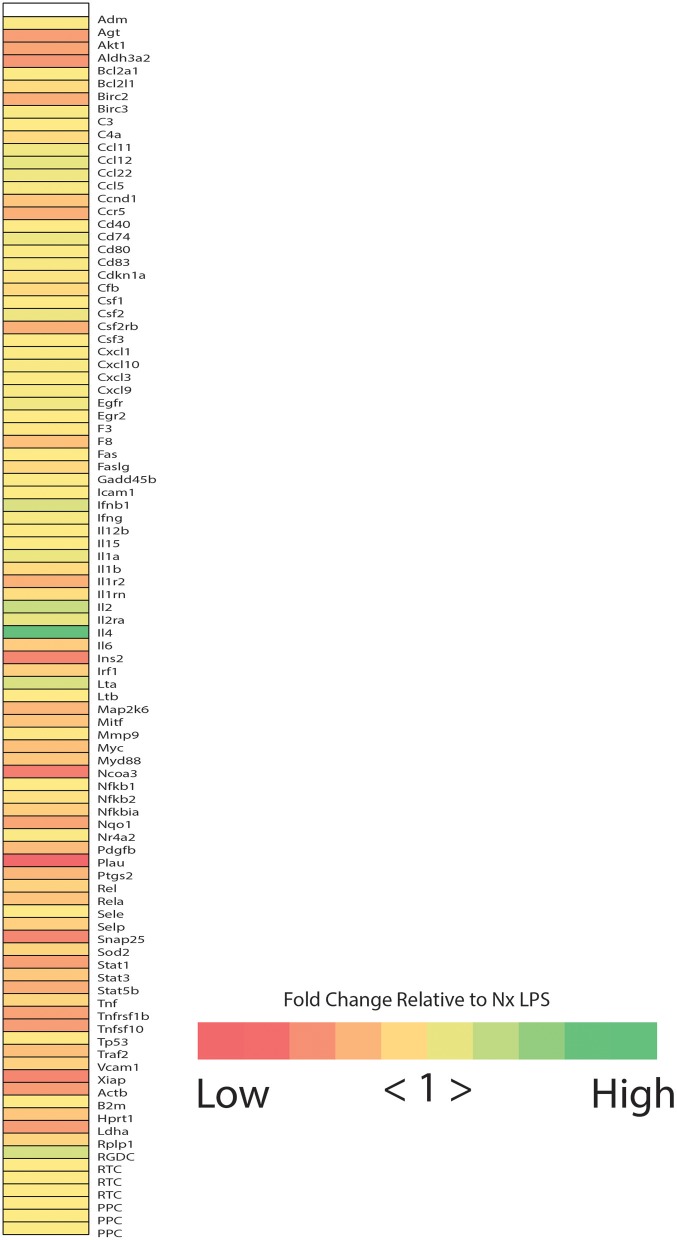
Neonatal IH increases the expression of NFκB-related cytokines. Heatmap represents the results of the Qiagen NF-κB signaling targets gene array. Colors represent the fold change between IH LPS and Nx LPS samples. “High” represents the highest fold change and “Low” represents the lowest fold change.

Based on observations in adult rats, we predicted that IH exposure would enhance microglial expression of classic pro-inflammatory cytokines/enzymes under basal and inflammatory (LPS) conditions. Prior to testing this prediction in all treatments and samples, we performed a preliminary screen for candidate genes altered by IH in whole brain microglia immunomagnetically isolated from LPS-treated pups exposed to 8 days of either Nx or IH, using the Qiagen NF-κB Signaling Pathway PCR Array (Figure 2). These preliminary results suggested that the most pronounced gene expression changes (with fold changes > 4 in IH LPS vs. Nx LPS) were not in classic inflammatory cytokines as we had originally hypothesized. Rather, the changes appeared to be in cytokines related to anti-inflammatory responses or T-helper cell responses, such as interferon-β (*Ifn*β*1*), interleukin-4 (*Il4*), and interleukin-2 (*Il2*). In further support of this anti-inflammatory effect in microglia isolated from the IH-exposed neonate, IH exposure tended to attenuate LPS-induced inflammatory gene expression instead of augmenting it (Figure 2). Thus, for the remaining experiments, we focused on genes identified in the array, in addition to the key inflammatory cytokines/enzymes identified from previous microglia research (11, 39).

**Figure 3:**
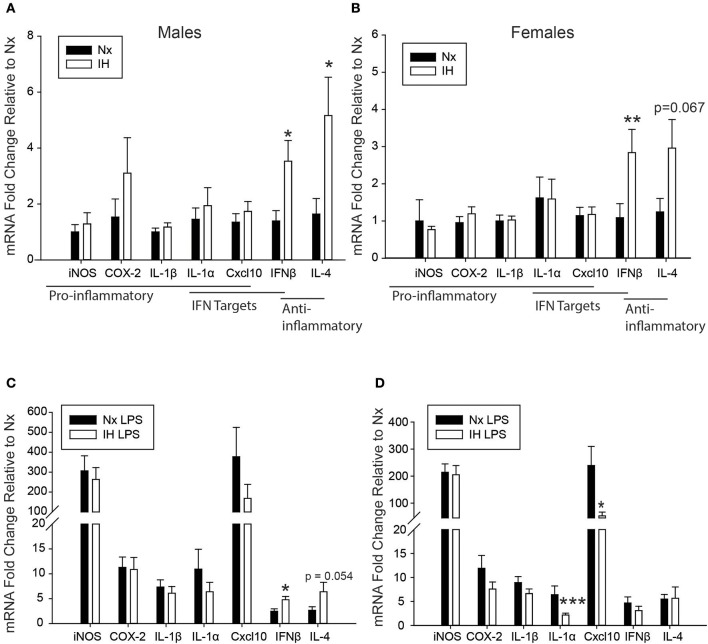
Neonatal IH augments anti-inflammatory cytokine and sex-specifically attenuates LPS-induced inflammatory cytokine gene expression. **(A,B)** Basal gene expression was analyzed in neonatal male **(A)** and female **(B)** microglia after 8 days of IH. **(C,D)** Microglial gene expression was analyzed in male **(C)** and female **(D)** pups exposed to IH followed by LPS challenge. Values represent average fold change +/– SEM *p < 0.05, **p < 0.01, ***p < 0.001 relative to respective Nx control, Two-way ANOVA.

We next tested the effects of neonatal IH on inflammatory gene expression in male (Figure 3A) and female (Figure 3B) microglia, as well as peripheral spleen macrophages (Supplementary Figure 2) following either vehicle or LPS treatment (Figure 3). In basal conditions (Figures 3A,B), there were no main effects of IH on male nor female neonatal microglial expression of the pro-inflammatory molecules *Inos, Cox-2*, and *Il1*β, nor the pro-inflammatory type I interferon targets *Il1a* and *Cxcl10* (Tables 2, 3; each table contains *F*-values, *p*-values, and *n* for all genes). Therefore, IH was not a strong stimulus for classic pro-inflammatory molecules. However, as expected, there was a main effect of LPS (Figures 3C,D) on each of these cytokines/enzymes (*p*-values for each gene in Tables 2, 3), indicating that neonatal microglia from these pups retain the capacity to respond to an inflammatory challenge. Surprisingly, IH appeared to have sexually dimorphic effects on the anti-inflammatory molecules *Ifn*β*1* and *Il4*. For males, there was a main effect of IH on *Ifn*β*1* (Table 2); *post-hoc* analyses showed that IH increased *Ifn*β*1* in both vehicle (*p* = 0.017) and LPS conditions (*p* = 0.012). There was a statistical trend for a main effect of LPS on *Ifn*β*1* expression in males (*p* = 0.071), but there was no interaction with IH (*p* = 0.961). In females however, while there was no main effect IH on *Ifn*β*1* expression (Table 3), there was a statistically significant effect of LPS (*p* = 0.007) and a significant interaction with IH (*p* = 0.016); *post-hoc* analyses showed a significant increase in *Ifn*β*1* only in vehicle conditions (*p* = 0.010), and not after LPS treatment (*p* = 0.407). *Il4* followed a similar trend to *Ifn*β*1*; in males, IH increased *Il4* (Table 2) in vehicle-treated animals (*p* = 0.012) and trended to increase it after LPS treatment (*p* = 0.054), but there was no main effect of LPS (*p* = 0.524), nor any interaction with IH (*p* = 0.643). In contrast, there was no main effect of IH on *Il4* in females (Table 3), although there was a main effect of LPS (*p* = 0.001) and a trending interaction with IH (*p* = 0.061). *Post-hoc* analyses for female *Il4* showed a statistical trend for IH to increase *Il4* in microglia from vehicle-treated pups (*p* = 0.067) but not from LPS-treated pups (*p* = 0.410).

**Table 2:** Statistical Values for P9 Males (Two-Way ANOVA).

		**Main effect IH**		**Main effect LPS**		**Interaction IH vs. LPS**	
**Gene**	**n/tx (Nx, Nx LPS, IH, IH LPS)**	***F***	***p***	***F***	***p***	***F***	***p***
*Inos*	10,9,9,8	0.162	0.690	504.092	< 0.001	0.427	0.518
*Il1β*	10,10,10,9	0.0000135	0.999	71.199	< 0.001	1.062	0.310
*Ifnβ1*	9,10,9,9	13.379	< 0.001	3.492	0.071	0.00237	0.961
*Il1α*	9,10,10,9	0.756	0.391	8.954	0.005	1.167	0.288
*Cxcl10*	10,10,10,8	0.0856	0.772	106.505	< 0.001	1.203	0.280
*Il4*	9,10,9,10	10.909	0.002	0.415	0.524	0.219	0.643
*Cox2*	10,10,10,9	1.188	0.285	49.052	< 0.001	1.647	0.208
*Stat3*	10,9,10,8	1.229	0.276	26.101	< 0.001	1.779	0.191

**Table 3:** Statistical Values for P9 Females (Two-way ANOVA).

		**Main effect IH**		**Main effect LPS**		**Interaction IH vs. LPS**	
**Gene**	**n/tx (Nx, Nx LPS, IH, IH LPS)**	***F***	***p***	***F***	***p***	***F***	***p***
*Inos*	8,8,8,8	0.296	0.590	625.399	< 0.001	0.396	0.590
*Il1β*	8,8,8,7	0.422	0.522	182.814	< 0.001	1.362	0.253
*Ifnβ1*	7,8,8,7	1.907	0.179	8.647	0.007	6.624	0.016
*Il1α*	8,8,8,7	4.165	0.051	6.623	0.016	4.053	0.054
*Cxcl10*	8,8,8,7	2.035	0.165	119.280	< 0.001	2.384	0.134
*Il4*	7,8,8,8	0.628	0.435	12.601	0.001	3.820	0.061
*Cox2*	7,8,7,8	0.0287	0.867	73.894	< 0.001	1.440	0.241
*Stat3*	8,8,8,8	0.0186	0.893	31.537	< 0.001	0.251	0.621

While there were no statistically significant interactions between IH and LPS for most inflammatory cytokines, *post-hoc* analyses of the main effects of LPS revealed that IH significantly attenuated the LPS-induced interferon targets *Il1a* (*p* = 0.009) and *Cxcl10* (*p* = 0.049) in females but not males (Figures 3C,D), corroborating the findings for *Ifnb1* and *Il4*. Interestingly, all these effects were specific to CNS macrophages, as these observations were not present in peripheral (spleen) macrophages (Supplementary Figure 2). There was no main effect of IH nor any interaction between LPS and IH in spleen macrophages from males (Supplementary Figure 2A) or females (Supplementary Figure 2B) at this developmental stage. Therefore, even though both male and female microglia had similar basal levels of IH-induced cytokine gene expression, IH attenuated female, but not male, microglial responses to a subsequent inflammatory LPS challenge, effects that were specific to the CNS.

Since 8 days of IH resulted in altered microglial cytokine expression, we further assessed if these changes occurred earlier in the IH time course. For these analyses, we exposed a separate cohort of pups to IH at P1 for 1 day only, and then challenged them (i.p.) with vehicle or LPS at P2. Microglia were assessed for cytokine expression (Supplementary Figures 2C,D). Although there was a main effect of IH on male *Ifn*β*1* expression (*p* = 0.027), *post-hoc* analyses only revealed a trend for increased *Ifn*β*1* expression in IH LPS conditions (*p* = 0.059). No other significant changes were identified for any cytokine in males or females. However, these results suggest that males may be more sensitive to IH effects at an earlier time point than females, and that *Ifn*β*1* may be one of the cytokines driving the effects of IH.

### Neonatal IH Sex-Specifically Changes Leukocyte Recruitment in the CNS

**Figure 4:**
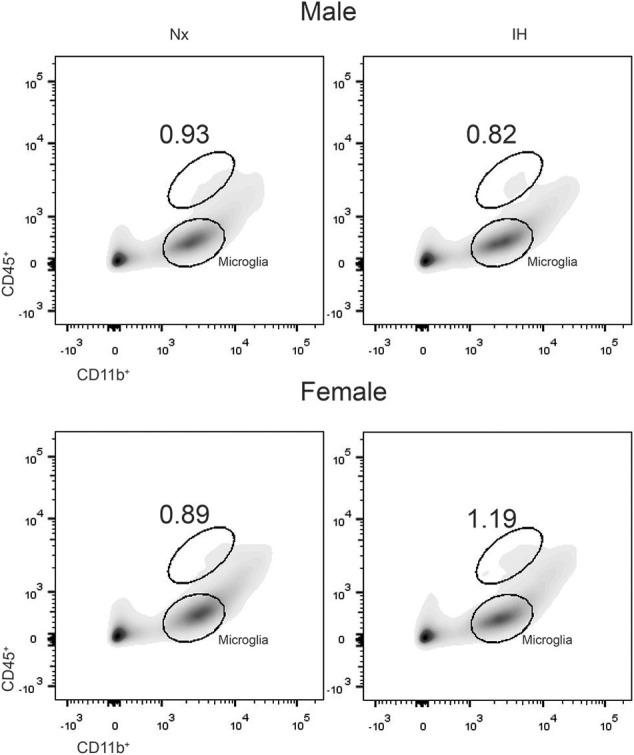
IH increases a novel population of CD11b^+^/CD45^high^ cells in females. Whole brains from males (Top) and females (Bottom) were dissociated into single cell suspensions and assessed by flow cytometry for CD11b and CD45 expression. Each panel shows a representative flow density plot for neonates exposed to 8 days of Nx or IH. The numbers represent the % of live cells gated.

**Figure 5:**
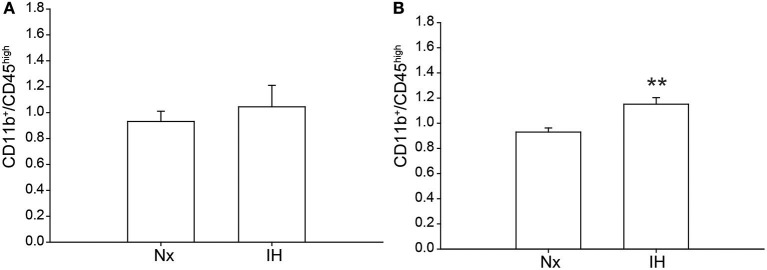
Quantification of the novel CD11b^+^/CD45^high^ population. Whole brains from IH or Nx-exposed neonatal males **(A)**; *n* = 6/treatment) and females **(B)**; *n* = 6/treatment) were assessed by flow cytometry for % of live cells expressing CD11b and CD45. Values represent the average % of live cells +/– SEM, **p < 0.001, *t*-test.

Previous studies demonstrated brain region-specific sex-differences in microglia number during early postnatal development (39). To determine if changes in microglia frequency and/or leukocyte recruitment could explain the sex-specific effects of IH on gene expression (Figure 4), we performed flow cytometry for CD11b, CD45, and CD4. Surprisingly, we found no main effect of IH (*F* = 0.290, *p* = 0.630) nor sex (*F* = 0.974, *p* = 0.335) on the frequency of CD11b^+^/CD45^low^ microglia in the whole brain. However, we found a leukocyte population (~0.8–1% of cells) that was CD11b^+^/CD45^high^ in both males (Figures 4, 5A) and females (Figures 4, 5B), which was augmented by IH only in females (*p* = 0.005) and not in males (*p* = 0.550). Because the antibody we used to label CD11b shares a common antigen with CD11c, it is possible that this population is comprised of monocyte-derived dendritic cells given the intermediate CD11b/c expression and their high CD45 levels. However, further analyses are needed to conclusively identify this leukocyte population.

**Figure 6:**
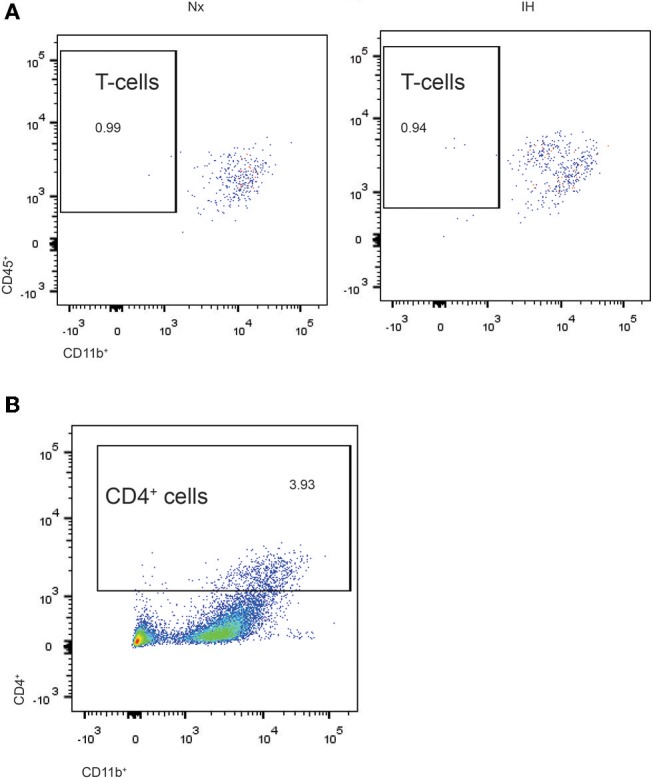
Flow cytometry analysis of CD4^+^ cells in whole brains isolated from neonatal rats. **(A)** CD4^+^ cells were gated and analyzed for concomitant CD45 and CD11b expression in Nx and IH conditions. T cells exhibit high CD45 with low CD11b expression. **(B)** Approximately 3–4% of total live cells were CD4^+^, the majority of which were high in both CD11b and CD45 expression; microglia are CD11b^+^/CD45^low^.

Given our gene expression analyses suggesting that microglia are increasing their expression of cytokines are associated with T-helper cell immunity (59, 60), we next tested whether the increases in *Ifn*β*1* and *Il4* expression corresponded to recruitment of CD4^+^ T-helper cells (Figure 6A). While we were able to detect a low frequency (~1%) population of CD4^+^/Cd45^high^/CD11b^−^ cells indicative of T cells, they did not differ by sex or treatment. Interestingly however, ~3–5% of CNS cells were CD4^+^, the majority of which were CD11b^high^ (Figure 6B). While very few of these cells were microglia (CD11b^+^/CD45^low^), it is established that CD4 labels monocytes in the rat (61), suggesting that CD4 may be a marker of bone-marrow derived macrophages/monocytes. The total number of CD4^+^ cells did not change by sex or treatment. Together, these findings demonstrate that CD4^+^ T-cell recruitment to the CNS is not altered by IH.

### Microglia RNA-Seq Reveals Sex-Differences in Basal and Inflammatory Conditions

**Figure 7:**
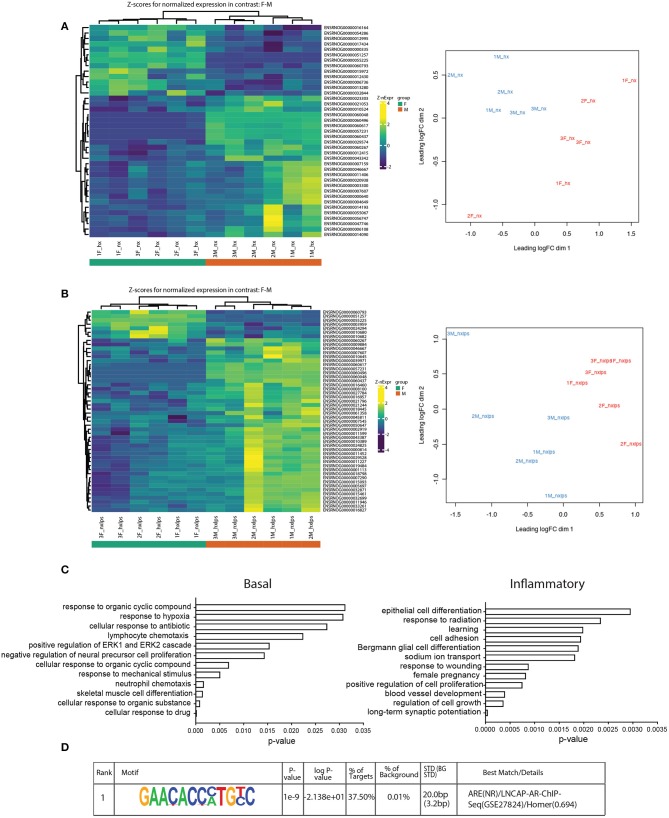
RNA-seq analyses of neonatal male and female microglia. **(A)** Heatmap of *z*-scores (left) and MDS analysis (right) for normalized gene expression in male and female microglia in basal conditions. **(B)** Heatmap (left) and MDS analysis (right) of normalized gene expression in male and female microglia in inflammatory (LPS) conditions. **(C)** Gene ontology for genes upregulated in males relative to females in basal (left) and inflammatory (right) conditions. **(D)** The top-ranking match for Homer motif analysis of the 8 genes identified in the GO category “cognition”.

The sex differences we identified in IH-induced gene expression and leukocyte recruitment may be indicative of gene pathways that are sexually dimorphic in either basal or inflammatory conditions, independent of IH exposure. Therefore, we used RNA-seq to assess sex differences in the neonatal microglial transcriptome after 8 days of IH exposure following vehicle or LPS treatment (Nx, IH, Nx LPS, IH LPS) (Figure 7). EdgeR bioinformatics analyses revealed that in basal (no LPS) conditions, 39 genes differed between males and females, independent of IH effects (Figure 7A, left). Males had more genes upregulated (than downregulated) relative to females. MDS analysis revealed that samples had high variability overall (Figure 7A, right), but clustered together based on sex, rather than IH treatment, suggesting that sex accounted for more of the variability between samples than IH treatment. Indeed, when analyzing vehicle-treated samples for an effect of IH, only a few genes were modified by IH in both males and females (Table 4).

**Table 4:** Differentially Expressed Genes from RNA-seq Results Analyzed in EdgeR.

**Treatment Comparison**	**Male Genes Up in IH**	**Male Genes Down in IH**	**Female Genes Up in IH**	**Female Genes Down in IH**	**Female NFκB Signaling Target Genes Down in IH**
Nx vs. Hx	ENSRNOG00000043342 (Gpr15l) ENSRNOG00000062158 (AC134224.3)	–	–	ENSRNOG00000029478 (Cyp4f39)	Not Applicable
Nx LPS vs. Hx LPS	ENSRNOG00000030616 (Slc25a45) ENSRNOG00000036674 (Cd7) ENSRNOG00000053494 (Mcpt1l1)	–	–	ENSRNOG00000046184 (RGD1561143) ENSRNOG00000004575 (Il1a) ENSRNOG00000019018 (Plat) ENSRNOG00000014524 (S1pr3)	ENSRNOG00000004575 (Il1a) ENSRNOG00000005731 (Birc3) ENSRNOG00000008525 (Csf3) ENSRNOG00000002802 (Cxcl1) ENSRNOG00000022256 (Cxcl10) ENSRNOG00000002525 (Ptgs2) ENSRNOG00000008144 (Irf1) ENSRNOG00000002723 (Sele) ENSRNOG00000022242 (Cxcl9) ENSRNOG00000018092 (Cd83) ENSRNOG00000020679 (Icam1) ENSRNOG00000010278 (Il6)

In LPS-treated samples (Figure 7B), 98 genes were differentially regulated between male and female pups. Similar to basal conditions, MDS analysis revealed that samples clustered based on sex and not IH treatment (Figure 7B, right). Further, when compared to females, more genes were upregulated in males than downregulated. Interestingly, although the effects of IH were not strong enough to reveal many significant differences with *n* = 3 biological replicates (and 10 million reads), the bioinformatics analyses did confirm that multiple LPS-induced inflammatory genes, including *Il1a*, were attenuated by IH in females, but not in males (Table 4 and Figure 3D). When we probed RNA-seq samples for the effect of IH on LPS-stimulated NF-κB signaling targets previously identified using the gene array, we found that multiple LPS-induced type I IFN genes were attenuated (Table 4) by IH pre-exposure in females only, confirming our previous findings.

Genes upregulated in males relative to females in both basal (Figure 7C, left) and inflammatory conditions (Figure 7C, right) were functionally annotated using DAVID for Gene Ontology (GO) class Biological Process. In basal conditions, males and females differed in genes related to the categories of “hypoxia,” “lymphocyte chemotaxis,” and “neutrophil chemotaxis,” observations that may contribute to the previous findings that certain leukocyte populations are sex-specifically recruited to the brain during IH. Additionally, several of the genes involved in chemotaxis, including the chemokine receptor Ccr3, were differentially expressed between males and females, as has been reported previously in the P4 rat brain (39).

To further examine relationships between all the genes that were significantly changed by sex irrespective of IH, we used STRING analysis of functional protein association networks (Supplementary Figure 3). The results showed two main gene networks: one involving cytokines/chemokines, and the other involving cellular proliferation/survival (Supplementary Figure 3A). These findings support the notion that IH-induced differences in cytokine expression (Figure 3) may be related to basal and IH-induced differences in leukocyte trafficking (supported by Figures 4, 5), and/or the ability of certain cell populations to proliferate.

Surprisingly, in inflammatory conditions (irrespective of IH), sex-specific changes were identified in gene categories related to “learning” and “long-term synaptic potentiation” (Figure 7C). Additional analyses examining Biological Process in Panther similarly revealed 8 genes enriched in the category “cognition”: Ptn, BDNF/NT-3, Dbi, Atp1a, Stra6, Fam107a, Agt, and Slc6a1 (Supplementary Figure 3B). Motif analyses of the promoters for these cognition-related genes revealed a common binding motif for the androgen receptor (Figure 7D), suggesting that the androgen receptor may play an important role in sex-related differences in neonatal susceptibility to cognitive deficits. Together, these results support the idea that sex-differences exist in P9 whole brain microglia in both basal and inflammatory conditions and suggest multiple candidate gene targets by which early life insults may change microglial function and impact cognition.

### Weight Gain in Young Adults Is Not Altered by Neonatal IH

**Figure 8:**
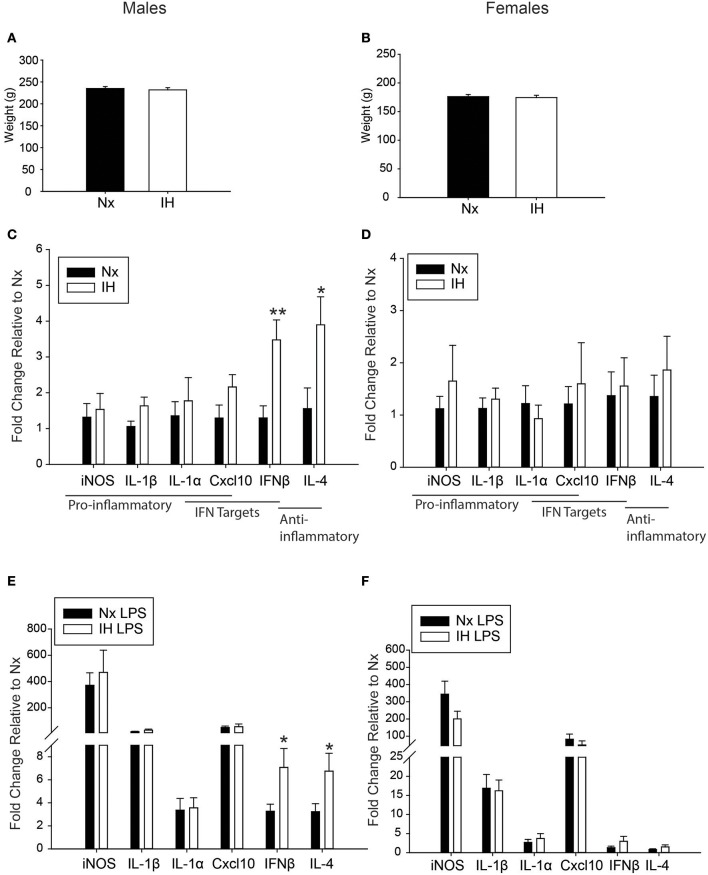
Neonatal IH augments anti-inflammatory cytokine gene expression in adult males. **(A,B)** Adult weights were unchanged in males **(A)** and females **(B)** 6 weeks following the last IH exposure. **(C,D)** Basal gene expression was analyzed in adult male **(C)** and female **(D)** microglia 6 weeks post neonatal IH exposure. **(E,F)** Microglial gene expression was analyzed in male **(E)** and female **(F)** neonates exposed to IH followed by LPS challenge. Values represent average fold change +/– SEM *p < 0.05, **p < 0.01, relative to respective Nx control, Two-way ANOVA.

Because weight changes observed acutely following neonatal IH exposure were previously reported to persist into adulthood (14), we also tested this in 6 week-old young adults that were neonatally exposed to 8 days of IH starting at P1 (Figures 8A,B). The average weight of adult males (Figure 8A) exposed to neonatal Nx (*n* = 21) was 235.14 g +/– 4.25 g and neonatal IH (*n* = 19) was 231.79 g +/– 5.16 g. Similarly, the average weight of females (Figure 8B) exposed to neonatal Nx (*n* = 14) was 176.14 g +/– 3.63 g and neonatal IH (*n* = 18) was 174.42 g +/– 3.97 g. There was no statistical difference in weight for males (*t* = 0.505, *p* = 0.616) nor females (*t* = 0.312, *p* = 0.757), suggesting that the paradigm of IH used in these studies does not exert effects on weight that persist into adulthood.

### Neonatal IH Sex-Specifically Disrupts Microglial Ifnβ1 and Il4 Expression Long-Term

We next assessed whether neonatal IH-induced changes in microglial cytokine gene expression were maintained long-term, into early adulthood. Six week-old males and females exposed to Nx or IH as neonates were injected with LPS (i.p. for 3 h), and microglia were isolated for gene expression analyses (Figures 8C–F). The majority of cytokines in adult males (Figure 8C and Table 5) and females (Figure 8D and Table 6) were unchanged in basal conditions, and 3 h of LPS was sufficient to increase the expression of most inflammatory cytokines. Interestingly, in adult males (but not females) there was a significant main effect of neonatal IH on *Ifn*β*1* expression (*p* < 0.001) and *Il4* (*p* = 0.003). *Post-hoc* analyses showed that IH augmented both *Ifn*β*1* and *Il4* expression in vehicle- (*Ifn*β*1 p* = 0.004; *Il4 p* = 0.020) and LPS-treated (*Ifn*β*1 p* = 0.020; *Il4 p* = 0.047) conditions. Thus, the effects of neonatal IH on male *Ifn*β*1* and *Il4* microglial gene expression persisted into adulthood, whereas those effects on female microglia did not. Surprisingly, while LPS significantly increased both *Ifn*β*1* and *Il4* in adult males, there was no effect of LPS on these cytokines in adult females (Table 6), despite the interaction between LPS and IH in neonatal females (Table 3). Together, these results support the idea that neonatal IH has sex-specific effects that persist into early adulthood in males, and that some responses to LPS differ developmentally.

**Table 5:** Statistical Values for Adult Males (Two-Way ANOVA).

		**Main effect IH**		**Main effect LPS**		**Interaction IH vs. LPS**	
**Gene**	**n/tx (Nx, Nx LPS, IH, IH LPS)**	***F***	***p***	***F***	***p***	***F***	***p***
*Inos*	7,9,7,9	0.0389	0.845	141.421	< 0.001	0.318	0.577
*Il1β*	7,9,7,10	1.090	0.305	74.278	< 0.001	0.146	0.705
*Ifnβ1*	7,10,7,9	15.610	< 0.001	12.239	0.001	0.640	0.430
*Il1α*	7,10,7,10	0.127	0.724	4.742	0.037	0.0151	0.903
*Cxcl10*	7,10,6,10	0.0253	0.875	54.175	< 0.001	2.518	0.123
*Il4*	7,10,7,10	10.283	0.003	5.028	0.032	0.305	0.585
*Stat3*	6,8,6,9	0.872	0.360	18.598	< 0.001	0.357	0.556

**Table 6:** Statistical Values for Adult Females (Two-way ANOVA).

		**Main effect IH**		**Main effect LPS**		**Interaction IH vs. LPS**	
**Gene**	**n/tx (Nx, Nx LPS, IH, IH LPS)**	***F***	***p***	***F***	***p***	***F***	***p***
*Inos*	6,7,6,7	0.421	0.523	388.729	< 0.001	1.732	0.202
*Il1β*	6,8,6,9	0.367	0.550	69.935	< 0.001	0.00204	0.964
*Ifnβ1*	6,8,6,8	0.0755	0.786	0.102	0.753	0.227	0.638
*Il1α*	6,8,6,8	0.162	0.691	5.597	0.026	0.525	0.476
*Cxcl10*	6,7,6,7	0.381	0.544	69.251	< 0.001	0.489	0.492
*Il4*	6,7,6,7	0.00872	0.926	0.321	0.577	0.0702	0.793
*Stat3*	6,8,6,7	0.449	0.509	21.908	< 0.001	0.0145	0.905

### Neonatal IH Does Not Impair Adult Working Memory

**Figure 9:**
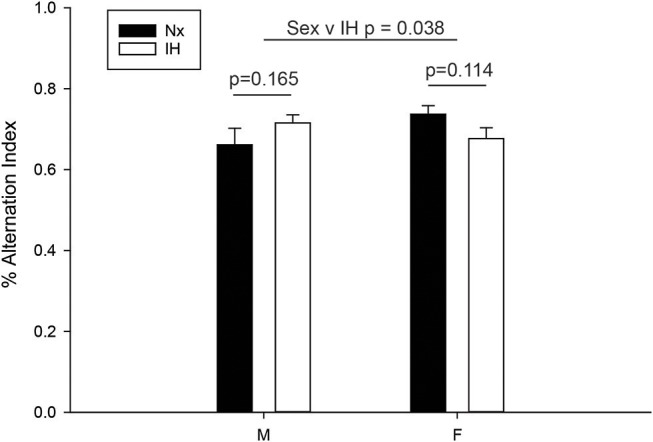
IH sex-specifically affects spontaneous alternation in naïve young adults. Behaviorally naïve adults who were exposed to Nx or IH as neonates were tested in the Y-maze spontaneous alternation task. Nx males (*n* = 9), IH males (*n* = 14), Nx females (*n* = 11) females and IH females (*n* = 12). The spontaneous alternation index is defined as the number of times a rat made three consecutively correct arm entries/total arm entries minus two. Results represent the average % alternation index in rats from three independent litters +/– SEM, Two-way ANOVA.

Since adult males exposed to neonatal IH demonstrated changes in the expression of microglial *Ifn*β*1* and *Il4*, cytokines with mixed effects on learning and memory (62–65), we next examined working memory in young adult (6–8 wk) male and female rats (Figure 9). Spontaneous alternation in behaviorally naïve rats was tested in a Y-maze, a frontal cortex and hippocampal-dependent working memory task (57, 58, 66). While there was a significant interaction between sex and IH treatment (*F* = 4.568, *p* = 0.038, Two-way ANOVA), *post-hoc* analyses identified no significant differences between the treatment groups. There was a statistical trend for better performance in females compared to males exposed to neonatal Nx (*t* = 1.879, *p* = 0.067), and a trending decrease in spontaneous alternation performance in neonatal IH-exposed females compared to neonatal Nx-exposed females (*t* = 1.613, *p* = 0.114); however, the effects were modest at best. Together, these results suggest that 8 days of neonatal IH does not strongly impair spatial memory in otherwise naïve animals, although there are sexual dimorphisms in the effects of IH on spatial working memory.

## Discussion

In this work, we initially hypothesized that neonatal IH would sex-specifically augment microglial inflammatory gene expression in both basal and inflammatory conditions, and that these effects would persist into adulthood. Contrary to our hypothesis, we found that neonatal IH augmented anti-inflammatory and type I interferon-related cytokines, and sex-specifically altered CNS myeloid populations, while attenuating LPS-induced inflammatory cytokine/chemokine expression acutely following IH. While these changes persisted into adulthood only in males, they were not associated with overall impairments in working memory nor alterations in spleen macrophage gene expression.

Our work also demonstrated sex differences in the effects of IH both acutely and long term. Acutely, neonatal IH increased anti-inflammatory cytokines in both P9 males and females, but only impaired LPS responses in P9 females. One intriguing explanation for the acute effects of IH on LPS responses in female vs. male microglia is a sex difference in the capacity to respond apoptotic CNS cells via phagocytosis (efferocytosis). Our results showed that IH increased CNS cell death by ~13–16% in both sexes. While we were not able to determine by flow cytometry if cell death was apoptotic (vs. necrotic), the lack of basal pro-inflammatory gene expression at both P1 and P9 following neonatal IH, and previous work in neonatal IH models (15), would support a non-inflammatory mechanism of cell death (i.e., apoptosis). It is well-established that macrophage efferocytosis is an anti-inflammatory process which can attenuate LPS responses (67–69). Since males and females exhibit differences in microglial phagocytosis in early postnatal life (43), female microglia may be more responsive to CNS apoptosis/cell death. Our RNA-seq data support sex differences in genes associated with apoptosis and efferocytosis; for example, the nuclear receptor Nr4a1, which contributes to the anti-inflammatory effects of efferocytosis in peripheral macrophages (70), is differentially expressed in our male and female microglia under basal conditions (Supplementary Figure 3). The idea that CNS cell death may account for the sex differences in LPS response would explain why changes in female LPS responses are acute, as IH-induced cell death would no longer be present in young adulthood and therefore would not induce an anti-inflammatory effect in microglia.

Another possible explanation for the acute effects of IH on LPS responses is the recruitment of other innate immune cells, which we predict is transient, but have not yet confirmed. By flow cytometry, we observed that IH sex-specifically increased a population of CD11b^+^/CD45^high^ cells that are likely to be other monocyte-derived cell types. Recent research in mice has identified a neonatal subpopulation of CD11c^+^ microglia that express CD4 and contribute to myelogenesis; it is possible that the myeloid population identified here has similar function (71). Alternatively, this myeloid cell population could be dendritic cells, which are important antigen presenting cells present in the choroid plexus under homeostatic conditions (72), but can also be derived from monocytes in the presence of IL-4 and IFNβ (73), two of the genes we find to be augmented by IH in the brain. Additionally, dendritic cells are strong producers of IFNβ (74). Thus, it remains unclear whether the changes we observe in *Ifn*β*1* and *Il4* gene expression are truly a reflection of alterations in microglial gene expression, or if they reflect responses to the unidentified alternative monocyte cell population. However, if the changes in *Ifn*β*1* and *Il4* are a consequence of changing CNS myeloid populations, it would suggest that males and females have different sources of these two genes, as only females exhibited a change in myeloid populations. Future work will need to investigate this possibility by characterizing the different myeloid populations identified for the first time here. Our RNA-seq data further supported the potential for a sex difference in the myeloid cell recruitment to the CNS, as a network of chemokine-related genes was differentially expressed (Supplementary Figure 3).

In addition to the sex differences in microglial gene expression following acute IH, there were multiple sex effects identified by RNA-seq that were independent of IH treatment. From the RNA-seq data, we observed changes in “cognition”-related genes in the context of inflammation, not in basal conditions. This is important given that OSA is often comorbid with other illnesses involving systemic inflammation. These results suggest that cognitive vulnerabilities may be unmasked because of infection. Motif analyses for DNA binding proteins that may be common regulators of these cognition genes identified a binding motif for the androgen receptor. Androgen receptors mediate inflammatory responses (75, 76) and CNS pathology (77), and androgens activate signaling pathways that influence the cognition-related genes identified in this study, including the GABA transporter Slc6a (78–81). Indeed, at least one study has even demonstrated that androgen receptors contribute to male-specific GABA_A_-mediated excitotoxicity in the hippocampus (79), which might underlie male vulnerability to cognitive dysfunction in early life. Thus, the microglial genes identified in this study may be useful candidates for future studies that investigate sexual dimorphism in cognitive dysfunction after neonatal stressors, such as IH.

Unlike females, males maintained their basal gene expression of anti-inflammatory cytokines acutely and long-term. Since males did not exhibit an acute increase in CNS myeloid populations, there is an as yet unidentified mechanism contributing to these long-term changes in *Ifn*β*1* and *Il4* expression in males. We posit that IH-induces modifications to the chromatin landscape, as has been reported for the carotid body (82). While the consequences of upregulated microglial cytokines in the male are unknown, type I interferons can inhibit synaptic plasticity associated with learning (83), and increased levels of IFNβ and IL-4 in the choroid plexus are associated with cognitive dysfunction in aging (62, 63). Although our results suggest that basal working memory is not impaired in young adults (6–8 wks) exposed to IH as neonates, it is unknown for how long the augmentation of these anti-inflammatory cytokines persists, or if they contribute to cognitive dysfunction in aging.

Our IH model recapitulates multiple aspects of other models of infant OSA, including SpO_2_ nadir to ~60% and poor weight gain (5). However, the severity of O_2_ desaturation caused by IH exposures in rats compared to infant OSA is questionable due to species differences in the oxygen reserve, suggesting that the 60%SpO_2_ nadir in neonatal rats is likely less severe than 60% in human newborns (84). Nonetheless, for experimentally modeling the effects of OSA, rats remain a good mammalian option (as compared to mice) since their oxygen dissociation curve is closer to that of humans (85). Other limitations include exposure of pups with dams during IH treatment, which others have suggested might reduce lactation, resulting in reduced neonatal weight gain (16); this has not been directly tested. Nevertheless, repeating these findings using a cross-fostering model which does not alter weight gain (16) may be beneficial to better understand the direct effects of IH on the pups independent of the mother. Lastly, the neonates were exposed to 15, uniformly distributed, hypoxic episodes/h, for 8 h periods during the light phase of each day. Since rat neonates do not establish diurnal/nocturnal patterns for wake/sleep until P16-P20 (86), this IH model does not necessarily recapitulate apneas experienced only during sleep. However, despite these constraints, the weight and SpO_2_ results we obtained are similar to those in other neonatal IH models used by others (14, 20, 38, 87).

Overall, the results presented here support the idea that IH is an early life stimulus that acutely modifies microglial function and induces long-lasting changes in microglial gene expression. Future studies will need to establish the functional consequences of IH-induced microglial gene changes in the context of long-term CNS inflammation and cognitive function.

## Data Availability

The sequencing datasets generated in this study are available in the NCBI GEO Datasets: GSE126946. All other data are presented in manuscript or additional files, and are available from the corresponding author upon reasonable request.

## Ethics Statement

This study was carried out in accordance with the recommendations of University of Wisconsin-Madison Institutional Animal Care and Use Committee. The protocol was approved by the University of Wisconsin-Madison Institutional Animal Care and Use Committee.

## Author Contributions

EK and JW prepared the manuscript and figures. EK collected and analyzed data for flow cytometry, PCR, and RNA-seq experiments. TW analyzed adult spleen samples. SC, AV, and MC performed behavioral experiments and analyses.

### Conflict of Interest Statement

The authors declare that the research was conducted in the absence of any commercial or financial relationships that could be construed as a potential conflict of interest.
