# Supplementary material for: Neonatal Intermittent Hypoxia Induces Lasting Sex-Specific Augmentation of Rat Microglial Cytokine Expression
Source: Front Immunol. 2019 Jul 2;10:1479. doi: 10.3389/fimmu.2019.01479 (PMC6615134; doi:10.3389/fimmu.2019.01479)
Supplement: Supplementary file 1 [file Data_Sheet_1.PDF]

Supplementary Material

Kiernan et al. Supplemental Fig 1

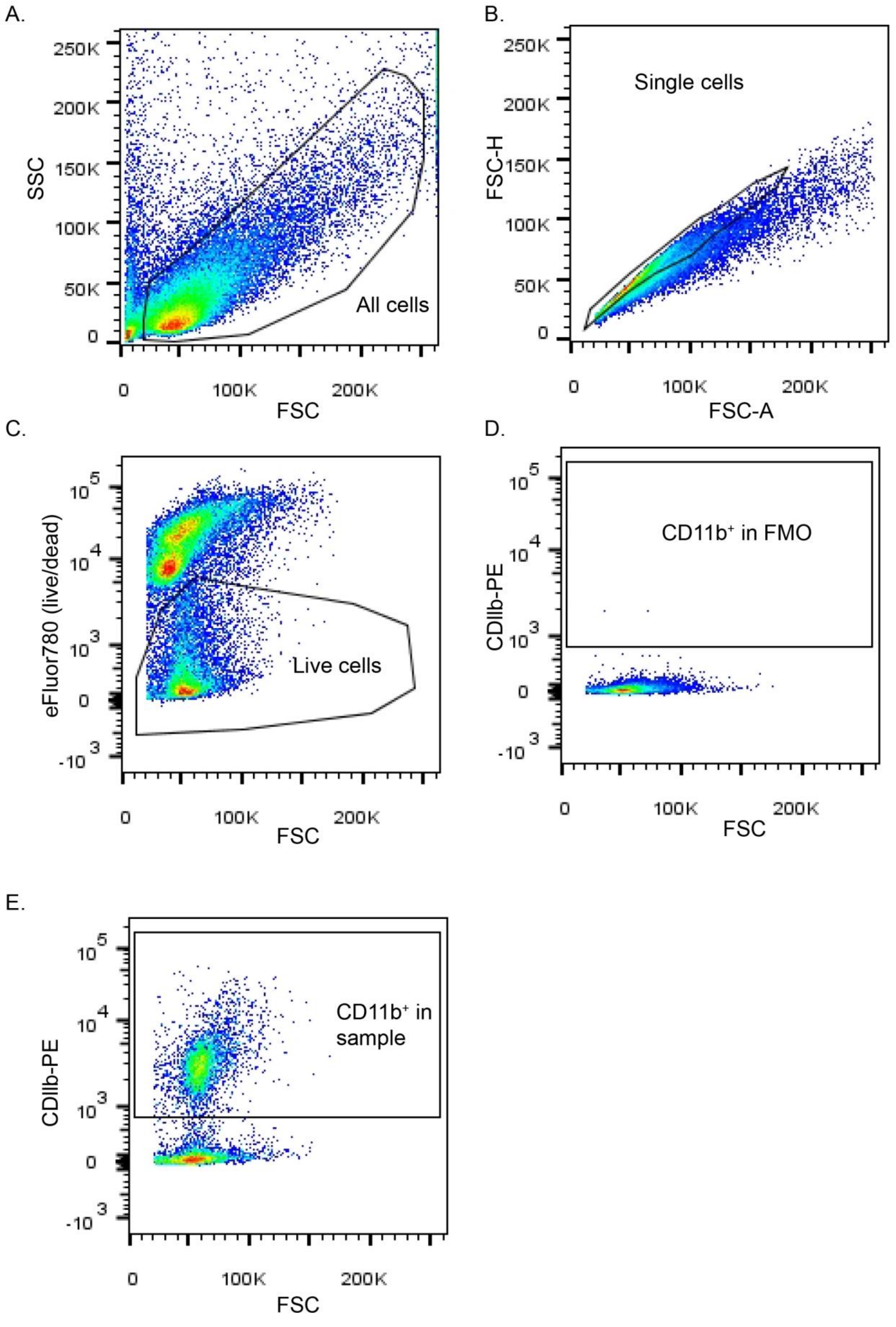

Supplementary Figure 1. Flow gating strategy. Total cells were gated using FSC-A/SSC-A (A) and then single cells were identified using FSC-A/FSC-H (B). Live cells were identified via eFluor780 exclusion (C). Fluorescence minus one (FMO) controls were used to set gates where positive cells would fall (D) and cells that were positive for any given fluorophore were identified in samples that expressed all fluorophores (E).

Kiernan et al. Supplemental Fig 2

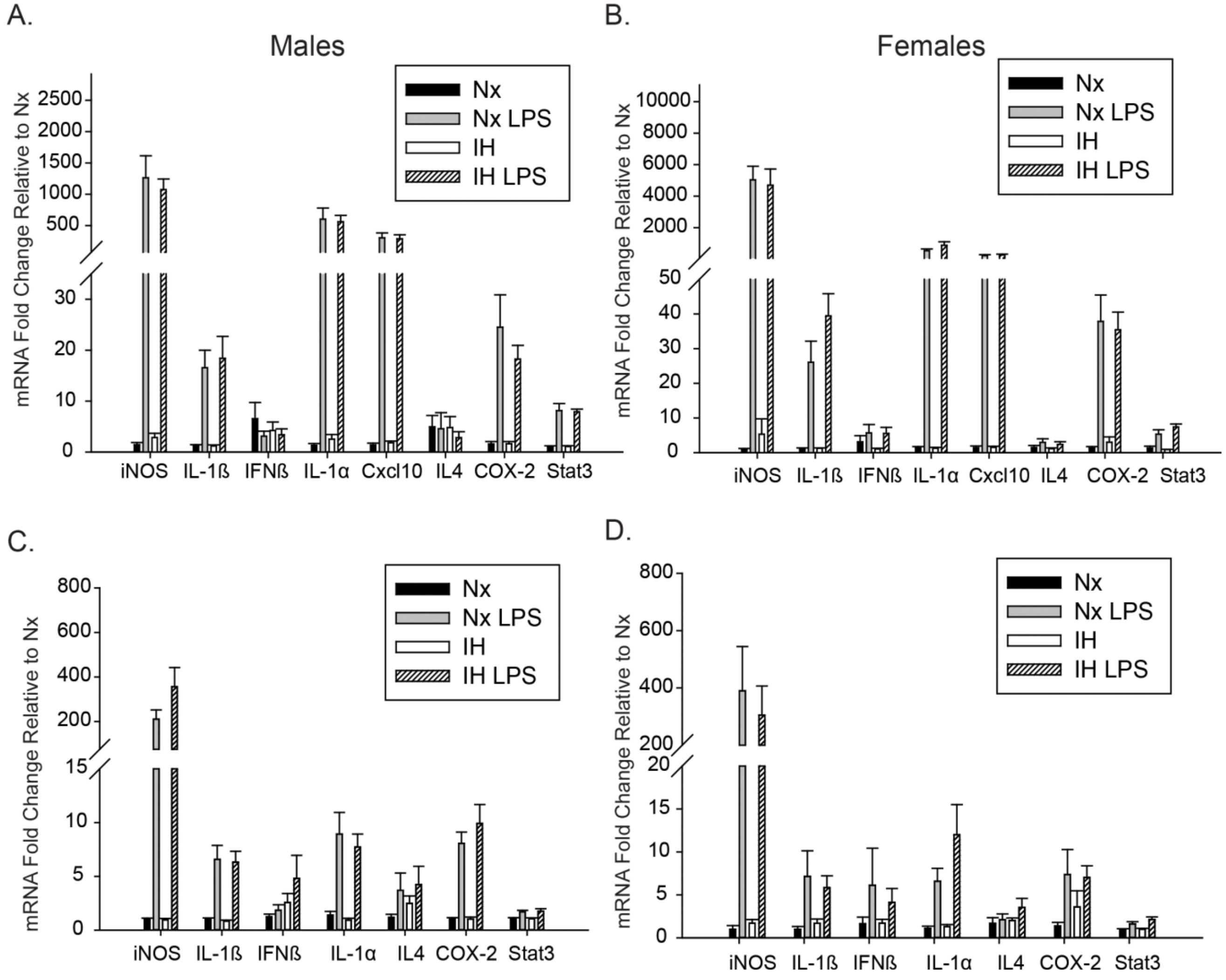

Supplementary Figure 2. Neonatal IH does not alter spleen macrophage gene expression at P9 nor microglia expression at P1. Spleen macrophages were isolated from males (A) and female (B) neonatal spleens following either Nx or IH exposure with vehicle or LPS injection. No significant effects of IH, nor interactions of LPS and IH were identified. Whole brain microglia from males (C; n = 9/treatment) and females (D; n = 5-7/treatment) were isolated after 1 day of IH exposure. A significant main effect was found for IH on male *Ifnβ* expression, $F_{1,31} = 5.436$, $p = 0.027$, Two-way ANOVA for IH v LPS. Post-hoc analyses revealed no significant differences between groups.

Kiernan et al. Supplemental Fig 3

A.

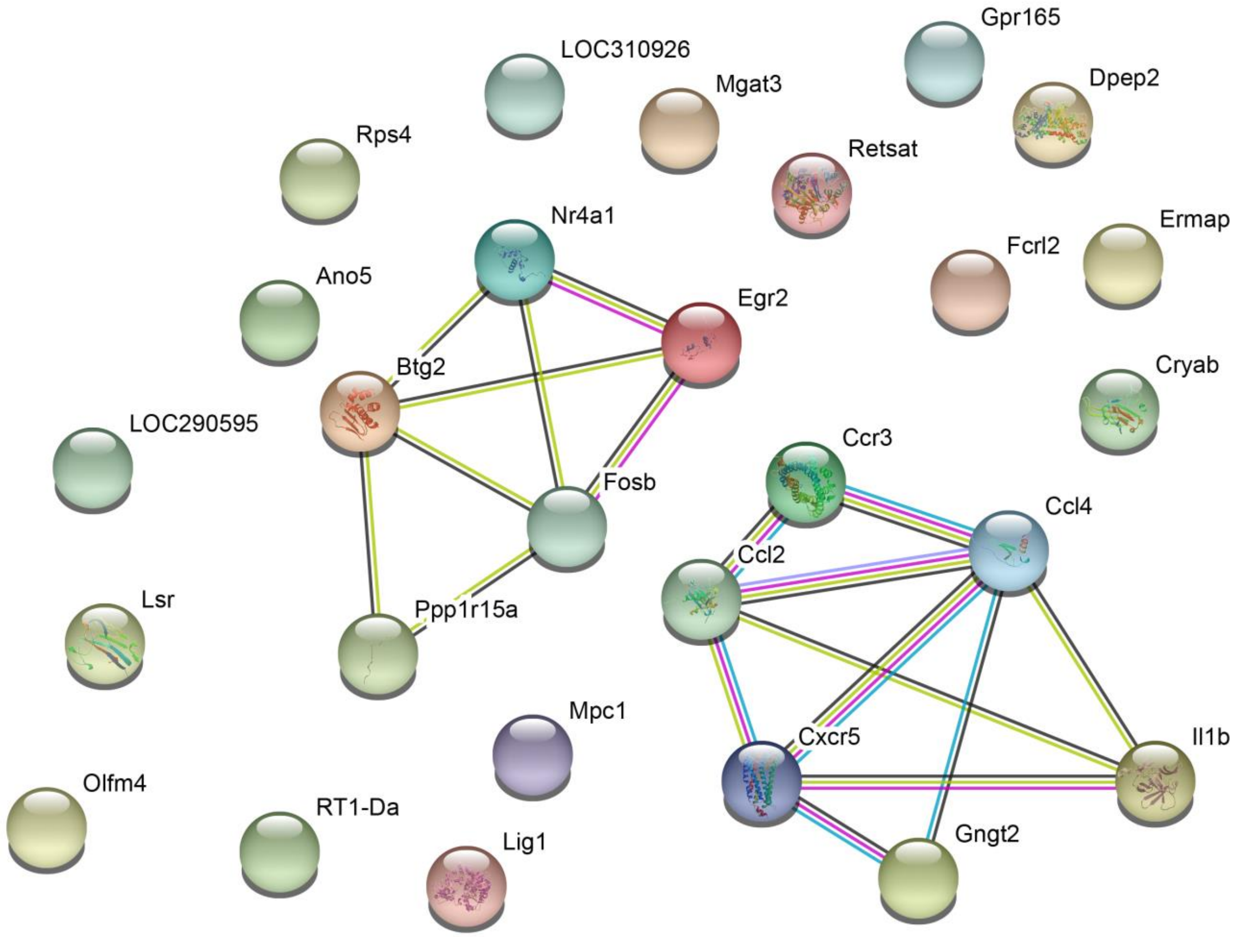

B.

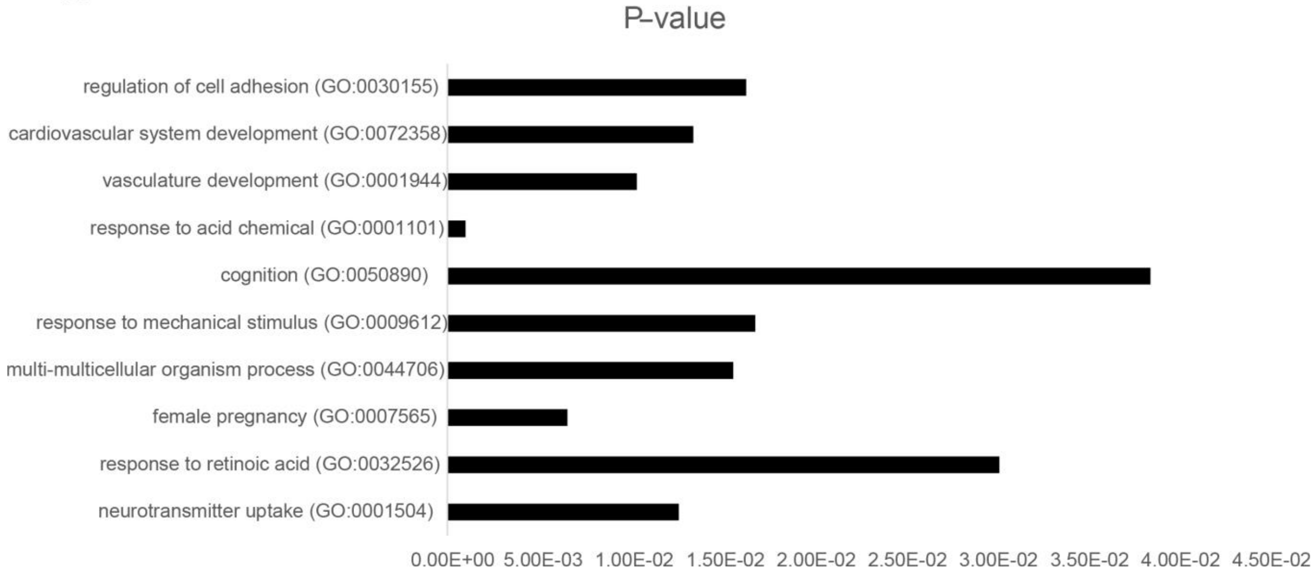

Supplementary Figure 3. Additional bioinformatics analyses on sex-specific genes. A) STRING network analysis on genes upregulated in males relative to females in basal conditions. B) Gene Ontology analyses for Biological Process performed using Panther on the list of genes upregulated in males relative to females in basal conditions.
